# Microbes as Key Players During Evolution: I From the Beginnings to the Appearance of Eukaryotes

**DOI:** 10.3390/ijms27198790

**Published:** 2026-09-30

**Authors:** Volker Schirrmacher

**Affiliations:** Immune-Oncological Center Cologne (IOZK), D-50674 Cologne, Germany; v.schirrmacher@web.de

**Keywords:** viroids, viruses, bacteria, archaea, bacteriophages, protists, endosymbiogenesis, transposon, prokaryote, eukaryote

## Abstract

Life on Earth originated in the Precambrian, around 4 billion years ago. This review focuses on microbes (viruses, bacteria, archaea) which developed in these prehistoric times and their roles during the later evolution of protists (eukaryotes). Among the different theories of life evolution, the author favors the theory of the RNA world as having been first. Over the past few decades, new molecular biological technologies and findings have changed the paradigm of mutation and selection as being the main driving force of biological evolution. Instead, microbes and microbe-derived genetic elements (e.g., plasmids, transposons) have been found to be capable of changing whole genomes and creating new species. Versatile RNA molecules have played important roles, such as regulating gene expression and affecting epigenetic changes, from primordial times up to the present. Microbes have been reported to show parasitic as well as mutualistic symbiotic relationships with their infected hosts. Examples of endosymbiogenesis are chloroplasts and mitochondria acting as new organelles, which facilitate photosynthesis and respiration within eukaryotic host cells. Considering the importance of these events, molecular mechanisms of photosynthesis and respiration are described in some detail in this review.

## 1. Introduction

Charles Darwin set out his theory of evolution in *On the Origin of Species* (1859) [1] (p. 198), in which he proposed that all organisms share a common ancestry and that the key mechanism of evolution is natural selection. In this review, common ancestry and natural selection are given full attention. Darwin reasoned that natural selection rests on three propositions. First, there is variation among organisms (within populations) in terms of their anatomical, behavioral, and physiological traits. Second, this variation is at least partly heritable, such that offspring tend to resemble their parents. Third, organisms with different variant forms are expected to leave different numbers of offspring in future generations. According to these ideas, natural selection is a creative process that generates novel features from the small individual variations that occur among organisms within a population [2] (p 14). The accumulation of such changes, Darwin argued, lead to the production of new organismal features and new species over long periods of time.

A look at the forms of life during different geological eras (Precambrian, Paleozoic, Mesozoic, Cenozoic) reveals periods of explosive evolution [1,2]. At the beginning of the Paleozoic, about 570 million years ago, there was an explosive expansion of phyla, with the evolution of external skeletons in animals, emergence of chordates, and an initial invasion of land by plants [1,2]. Major extinctions also occurred 445, 375, 251, and 200 million years ago. The almost complete extinction of dinosaurs 66 million years ago at the end of the Mesozoic was followed by diversification into birds (derivatives of dinosaurs), mammals, and modern plants in Cenozoic times (about 65 million years ago) [1,2,3]. These examples suggest that there may be more at work during evolution than small variations.

In this review, microbes include microscopic small living beings such as viroids and viruses, prokaryotes (bacteria, archaea), and eukaryotes. The first part of this review covers the period from the beginnings to the origin of eukaryotes. A second part will deal with the evolution from unicellular to multicellular organisms.

## 2. Key Points in the Evolution of Life

This section first describes the Earth’s ancient anaerobic environment. The development of life is then described in distinct steps; namely, chemistry and biochemistry, the RNA world, energy and metabolism, genetic programs, and structure and biodiversity.

### 2.1. Earth’s Prehistoric Environment

About 4.1 to 3.8 billion years ago, the Earth experienced a meteor bombardment [4]. After cooling, violent storms raged, with heavy rains accompanied by lightning and the release of electrical energy. Meanwhile, widespread volcanism spewed molten rock and boiling water from beneath the Earth’s surface [2] (p. 27). This Archean world was without oxygen (i.e., anaerobic). The atmosphere in these times consisted of N_2_ (from volcanic eruptions), H_2_O (as steam), CO_2_, CH_4_, H_2_S, NH_3_, CO, and other gases. Starting about 2.7 billion years ago, the O_2_ liberated via oxygenic photosynthesis on the ocean’s surface oxidized the iron dissolved in the oceanic water, which became insoluble and accumulated on the ocean floor. These enormous and long-lasting processes are evidenced by the banded iron formations (BIFs) of sedimentary rocks, composed of alternating bands of iron-rich minerals and silica (SiO_2_)-rich chert. Over time, the Earth’s atmosphere also changed. The optimal percentage of atmospheric oxygen for cyanobacteria was (and still is) about 10% [2] (p 54); at present, the atmosphere actually contains about 20% oxygen. The other gases are N_2_ (78%), CO_2_ (0.04%), NH_3_ (0.00019%), and noble gases. O_3_ (ozone) exists primarily in the stratosphere, 15–50 km above the Earth’s surface.

There are a number of hypotheses in the scientific community regarding the question of how life on Earth started. Some scientists postulate that Mars has exerted an influence [4,5], or that meteoric impacts potentially produced prebiotic compounds such as cyanosulfides [4]. Other scientists put minerals and proteins at the beginning, while others argue that there must have been metabolic circles (based on proteins or minerals) in the very beginning. The author of this review favors the view that, in the beginning, there was an RNA world which gradually diversified into a world with RNA, proteins, and DNA.

Evolution most likely started with prebiotic synthesis of small organic molecules made up of carbon (C), hydrogen (H), nitrogen (N), sulfur (S), and phosphorus (P), followed by the formation of polymers. Unique organic chemicals such as nucleic acids, proteins, carbohydrates, and lipids were key players in this process. These polymers were protected from the outside world (e.g., seafloor hot springs) by semi-permeable lipid membrane vesicles [1,2,3].

The evolution of bioenergetics provides a link between Earth’s history and that of life on Earth [4]. To deepen our knowledge of microbial evolution, a promising strategy is to combine large-scale comparative phylogenetic analyses of genes related to physiology with experimental data [6].

### 2.2. The RNA World

The primordial RNA world evolved about 3.8 billion years ago. Versatile RNA molecules used the semi-permeable lipid membrane vesicles as a “playground” and, over time, developed—among other things—functional self-cleaving ribozymes and defensive CRISPR/Cas complexes. Ribozymes have become the essential elements for protein synthesis of cellular organisms as part of ribosomes, with ribosomal RNA (rRNA) distinguishing the major trees of life, as follows: bacteria, archaea, and eukaryotes [2]. The evolution of ribosomes, messenger RNA (mRNA), aminoacyl transfer RNA (tRNA), and translation was a prerequisite for the production of proteins (i.e., polymers consisting of amino acids). Ribozymes also play roles in many modern cellular processes, such as self-splicing introns, ribonuclease P (an essential endonuclease for tRNA maturation), virus genomes, and replication of telomeres (eukaryotic chromosome ends) [3]. CRISPR/Cas is an ingenious antiviral defense system in prokaryotes [7], which contains a unique type of repetitive DNA stretches (repeats) termed clustered regulatory interspaced short palindromic repeats (CRISPRs). These repeats are interspersed with highly variable sequences, referred to as spacers, which originate from either plasmids or phages and comprise the “immunological memory” of prokaryotes [5]. CRISPR-associated (Cas) genes encode conserved proteins with endonuclease activity that, together with CRISPR, make up the CRISPR/Cas antiviral defense system in prokaryotes (Figure 1).

### 2.3. Energy and Metabolism

The plasma membranes of prokaryotic cells evolved with the enzyme ATP synthase, producing adenosine triphosphate (ATP) without requiring energy from ATP. These cells also evolved proton gradients and electron transport chains (ETCs). ATP had established its central role in microbial bioenergetics very early on, with ATP as an energy source and the rotor/stator enzyme ATPase appearing universally in living cells [6]. It has been proposed that life is driven by a chemical reaction that produces ATP, where ATP maintains the life process far from equilibrium [6].

Important coenzymes of ETCs were (and still are) nicotinamide adenine dinucleotide (NAD) and its phosphate (NADP), coenzyme A (CoA), and flavin adenine dinucleotide (FAD). Inorganic and organic compounds serve as electron donors. The final electron acceptors of the ETC mainly include nitrate, sulfate, fumarate, carbon dioxide (CO_2_), and oxidized metals [1,2,3,8].

Prokaryotic respiration involves the oxidation of sugars via glycolysis and fermentation with lactate or ethanol as end products, as well as byproducts such as hydrogen sulfite (H_2_S) or methane (CH_4_). This metabolic process, combined with the ETC and ATP synthase, enabled the production of ATP—the unique and universal energy source (“currency”) of living cells. Most free energy in ATP resides in the triphosphate group, especially in two phosphoanhydride bonds between the three phosphate groups. These two bonds are called “high-energy bonds,” as a great deal of free energy (7.3 Kcal/mol) is liberated from these bonds when ATP is hydrolyzed by ATPases to adenosine diphosphate (ADP) or adenosine monophosphate (AMP) and inorganic phosphate (P_i_) [2]. This high energy results from the repulsion between closely packed, negatively charged oxygen atoms in adjacent phosphate groups which, upon breaking of the bonds, is released. Energy can be released not only from ATP, but also from other molecules such as GTP, CTP, UTP, and phosphocreatine.

ATP is neither a fuel nor a source of energy, and is formed as it is needed; for instance, by oxidative processes in mitochondria [2] (p. 61). ATP is an intermediate in a coupled reaction of two enzymes [2] (p. 61) and takes part in a great variety of cellular events, including the flick of a flagellum, the streaming of cytoplasm, the biosynthesis of organic molecules, and the active transport of molecules across the plasma membrane [1].

**Figure 1:**
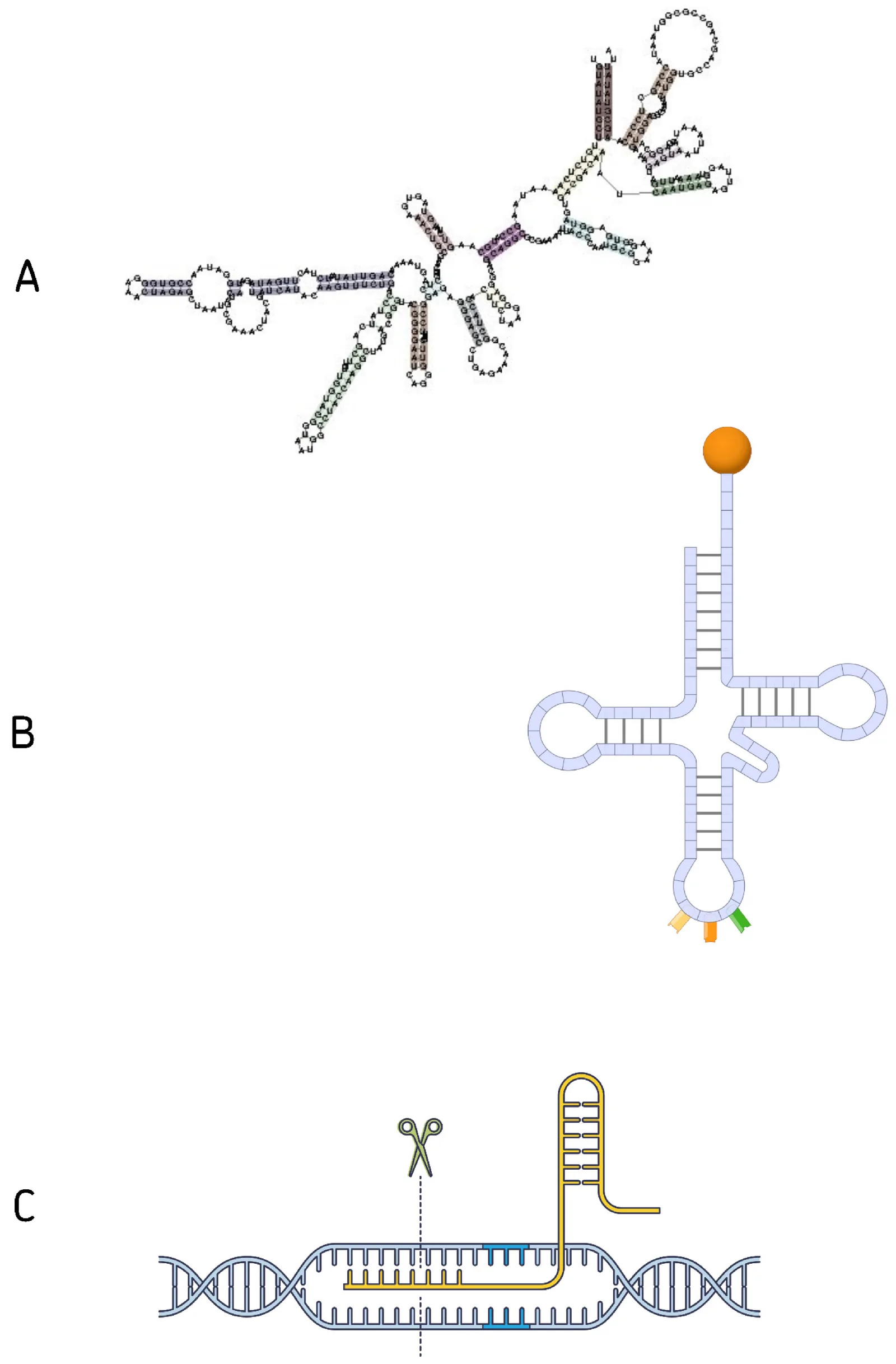
Versatile RNA molecules. Ribosomal RNA (rRNA, **A**) and transfer RNA (tRNA, **B**), together with ribosomes, make up the machinery for protein synthesis. Secondary structures are shown: (**A**) the 5′-domain of rRNA with its characteristic loops; (**B**) tRNA with its binding site for a distinct amino acid and its anti-codon (complementary to that of the mRNA); (**C**) the CRISPR/Cas system. (**A**) is reproduced from Wikimedia Commons under the respective Creative Commons or Public Domain licenses. (**B**,**C**) are based on content licensed from iStock and Shutterstock.

### 2.4. Genetic Programs

In the RNA world [9], ribonucleic acid was both the genotype and phenotype. RNA consists of 4 nitrogenous nucleotides; namely, purine bases (adenine (A) and guanine (G)), pyrimidine bases (cytosine (C) and uracil (U)), sugar (ribose), and phosphoric acid. A polynucleotide chain is built of a “backbone” of phosphoric acid and ribose molecules, where each sugar holds a nucleotide base. Replication of up to 100 nucleotides in RNA is possible via base pairing (e.g., A-U and G-C). Modifications of RNA have been explored in infectious non-coding circular RNAs [10]. Once a translation system and a genetic code evolved, proteins rapidly took over most catalytic functions for metabolic activity. Proteins have a more complex alphabet (20 different amino acids) than RNA (4 nucleotides), and thus can form a more varied array of polymers. For example, no RNA molecule has been found that can carry out redox reactions similarly to proteins such as oxidoreductases or break carbon/carbon bonds similarly to lyases [10]. As RNA ceased to be the exclusive domain of the genotype and phenotype, evolutionary forces may have begun to select for new genetic molecules and, eventually, DNA replaced RNA as the genome [10]. DNA differs from RNA in that it contains thymine (T) instead of U and 2-deoxyribose instead of ribose. Perhaps the RNA-to-DNA conversion happened after proteins such as reverse transcriptase (RT) were developed [10]. In modern organisms, deoxyribonucleotides are synthesized from ribonucleotides using the enzyme ribonucleotide reductase, supporting the view that RNA developed first [10]. The lower reactivity of DNA made it a more stable carrier of genomic information [10]. Complementary base pairing in DNA (A-T and G-C) occurs via multiple hydrogen bonds, and the sugar phosphate “backbones” keep the double helix at a constant diameter for the entire length of the molecule. Natural selection, as proposed by Darwin, made sure that these innovations were maintained.

Although genetic information is coded in the nucleotide base sequence inside the double-stranded DNA (dsDNA) molecule [2], DNA itself does not participate directly in protein synthesis. As an intermediary, another nucleic acid evolved, as follows: mRNA. Triplet codes in DNA are transcribed into mRNA by RNA polymerase, with U substituting for T [2]. Only one strand of dsDNA acts as a coding strand for mRNA. Prokaryotes organize their DNA genome of 1–5 million base pairs (bp) into a single circular dsDNA chromosome without histones. These chromosomes are highly compacted through supercoiling, and are organized into a double helix with independent domains and associated proteins. The chromosomes are located in non-membrane-bound cytoplasmic regions called the nucleoid. They replicate with dichotomy, not through mitosis [1,2]. In addition, prokaryote cells contain replicons; in particular, circular dsDNA plasmids.

The chromosomes and plasmids of prokaryotes differ in size (large versus small; 1 million bp versus 1000 bp), in function (essential genes for survival and reproduction versus non-essential genes; e.g., those conferring antibiotic resistance), and in the mode of gene transfer (vertical (inherited through division) versus horizontal). Chromosomes and plasmids replicate independently. There is only one chromosome, while the number of plasmids can be greater than one.

In contrast to bacteria, archaea possess a chromosome replication system resembling that of eukaryotes [11]. This was demonstrated for *Sulfolobus acidocaldarius*, an archaea isolated in 1972 from a Yellowstone hot spring [11]. Studies in this model organism for archaeal biology have improved our understanding of archaeal chromosome organization, DNA replication and segregation, cell division, metabolism, protein glycosylation, assembly and regulation of archaeal surface structures, and biofilm formation [11].

### 2.5. Structure and Biodiversity of Microbes

Examples demonstrating the structural and biological diversity of microbes are shown in Figure 2.

So far, this section has described—among other things—the structure and biodiversity of RNA molecules. For the author, a logical extension is to show also the structure and biodiversity of viruses, bacteria, and archaea.

### 2.6. From FUCA to LUCA, LACA, and LBCA

Major evolutionary transitions were required before eukaryotic cells could appear. Once basic biomolecules had formed and become abundant, the first transition was proposed to have led to the first universal common ancestor (FUCA), composed of self-replicating RNAs which were capable of catalyzing the binding of amino acids [12]. From the age of FUCA (about 4.2 billion years ago), the next transition was that to the age of progenotes. This transition involved the duplication and recombination of proto-genomes, leading to specialization in protein production and the exploration of protein/metabolite interactions in the prebiotic soup [12]. Multipotent communities with rudimentary genetics and horizontal gene transfer facilitated probiotic enzyme reactions and metabolic pathways. The last universal common ancestor (LUCA) (about 4.2 billion years ago) of ribosome-encoding organisms [13] was a progenote able to perform the basic metabolic functions of a cell, but still unable to interact with lipids [14]. Evidence indicates that the evolution of lipid interaction pathways occurred at least twice, resulting in the development of bacterial-like and archaeal-like membranes [11]. The last archaeal common ancestor (LACA) and the last bacterial common ancestor (LBCA) branched from the LUCA about 4–3.5 billion years ago [13,14].

The evolution of 11,272 gene families has been studied to identify the root, extent of gene transfer, and nature of the LBCA [15], with the results suggesting that the LBCA was a free-living flagellated rod-shaped double-membraned organism. Overall, two-thirds of gene transmissions have been vertical, suggesting that a rooted tree provides a meaningful frame of reference for interpreting bacterial evolution [15]. In another study, 146 protein families were studied with respect to the LBCA, belonging to the conserved predicted core of its metabolic network. The results indicated that the LBCA performed gluconeogenesis for the purpose of cell wall synthesis and harbored the acetyl-CoA pathway [15].

## 3. Viroids and Viruses

### 3.1. Viroids

Viroids formed in the primordial RNA world about 3.8 million years ago. They are the smallest known self-replicating circular RNA pathogens. The viroid genome is a circular single-stranded (ss) non-coding RNA with a compact secondary structure [16]. A naked plant-specific RNA viroid has been found to be tenfold smaller than the smallest known viral RNA [16]. Viroids share many properties with ribozymes but the latter are smaller [17], with viroid RNA ranging in length from 246 to 434 nucleotides while that of ribozyme RNA is 50 to 150 nucleotides. Viroids contain a conserved central region, while ribozymes consist of a core and three helices [17].

Sequence analyses have revealed that viroid-like RNAs (capsidless selfish elements) have evolved into viruses [18]. During evolution, viruses or their ancestors are assumed to predate the appearance of the last universal cellular ancestor (LUCA), considering its complex virome [19].

Viroids comprise two families, one of which harbors an enzymatically active hammerhead motif, while the other family relies on host factors. At present, viroids cause diseases in plants such as potato, avocado, or citrus, and replicate in chloroplasts or the nucleus [16]. They presumably cause diseases via viroid-derived small inhibitory RNAs (RNAi), causing transcriptional or post-transcriptional silencing of important host genes [20].

### 3.2. Theories of Viral Eukaryogenesis and Virus-Mediated Horizontal Gene Transfer

The viral eukaryogenesis (VE) hypothesis [21] proposes that eukaryotes originated as an emergent superorganism and thus did not evolve from the LUCA via descent with incremental modifications. According to the VE hypothesis, the eukaryotic nucleus descends from a viral factory, the mitochondrion from alpha-proteobacteria, and the cytoplasm and plasma membrane from an archaeal host [21]. A very recent study reported compelling evidence for multiple waves of horizontal gene transfer (HGT) from diverse bacterial donors [22], shaping the proteomes of pre-LECA ancestors. Nucleocytoviricota viruses apparently played a facilitating role in these HGT processes [22]. Some waves of HGT likely preceded mitochondrial endosymbiosis [22]. These studies suggest that, early on, viruses affected eukaryogenesis.

### 3.3. Viruses

It has been suggested that viruses are essential agents within the roots and stem of the tree of life [23]. Life most likely originated in oceans, and ocean viruses are the most common biological entities by an order of magnitude [18]. A genomic analysis of two uncultured marine viral communities revealed 374 and 7114 viral types [24].

#### 3.3.1. RNA Viruses

*Retroviridae* are a family of viruses that reverse-transcribe their RNA genome and integrate the resulting complementary DNA (cDNA) into the genome of the host cell. The International Committee on Taxonomy of Viruses currently lists 65 species of retroviruses [25]. Retroviruses and transposable elements (TEs) have been key players in evolution from prehistoric times to the present. TEs are discrete pieces of DNA that can move from one site to another within (and sometimes also between) genomes. They can be considered as widespread genetic parasites that copy and insert themselves “selfishly” across host genomes. TEs can also drive evolutionary dynamics and genetic diversity. Figure 3 shows structures from retroviruses and the life cycle of an RNA retrotransposon.

#### 3.3.2. DNA Viruses

DNA viruses have highly diverse and often exceptionally complex morphotypes [26]. DNA viruses of archaea are fundamentally different in morphology and genome from the DNA viruses of bacteria and eukaryotes [26].

The discovery of a giant DNA virus of the protist *Amoeba* in 2003 changed the field of virology [27]. It initiated a reappraisal of the upper limits of the viral world in terms of particle size (>400-nanometer (nm), visible via light microscopy) and genome complexity (2.5 megabases > 1000 genes) [28]. Extended studies of giant viruses of amoebae revealed that they have genetic, proteomic, and structural complexities that were not thought to exist among viruses and that are comparable to those of bacteria, archaea, and small eukaryotes [27]. Giant virus particles contain mRNA and more than 100 proteins. Their gene repertoires are broader than those of other viruses, with some genes encoding translation components catalyzing the binding of tRNA to the ribosome, including (i) four amino-acyl tRNA synthetases; (ii) peptide release factor 1; (iii) elongation factor thermo unstable; and (iv) translation initiation factor 1 [29].

Giant viruses of amoebae include mimiviruses, Marseilleviruses, Pandoraviruses (Figure 2C), Pithoviruses, Faustoviruses, and Molliviruses [27]. Giant viruses, due to their similarities to eukaryotic cells, could have played a role in the development of eukaryotic cells.

**Figure 3:**
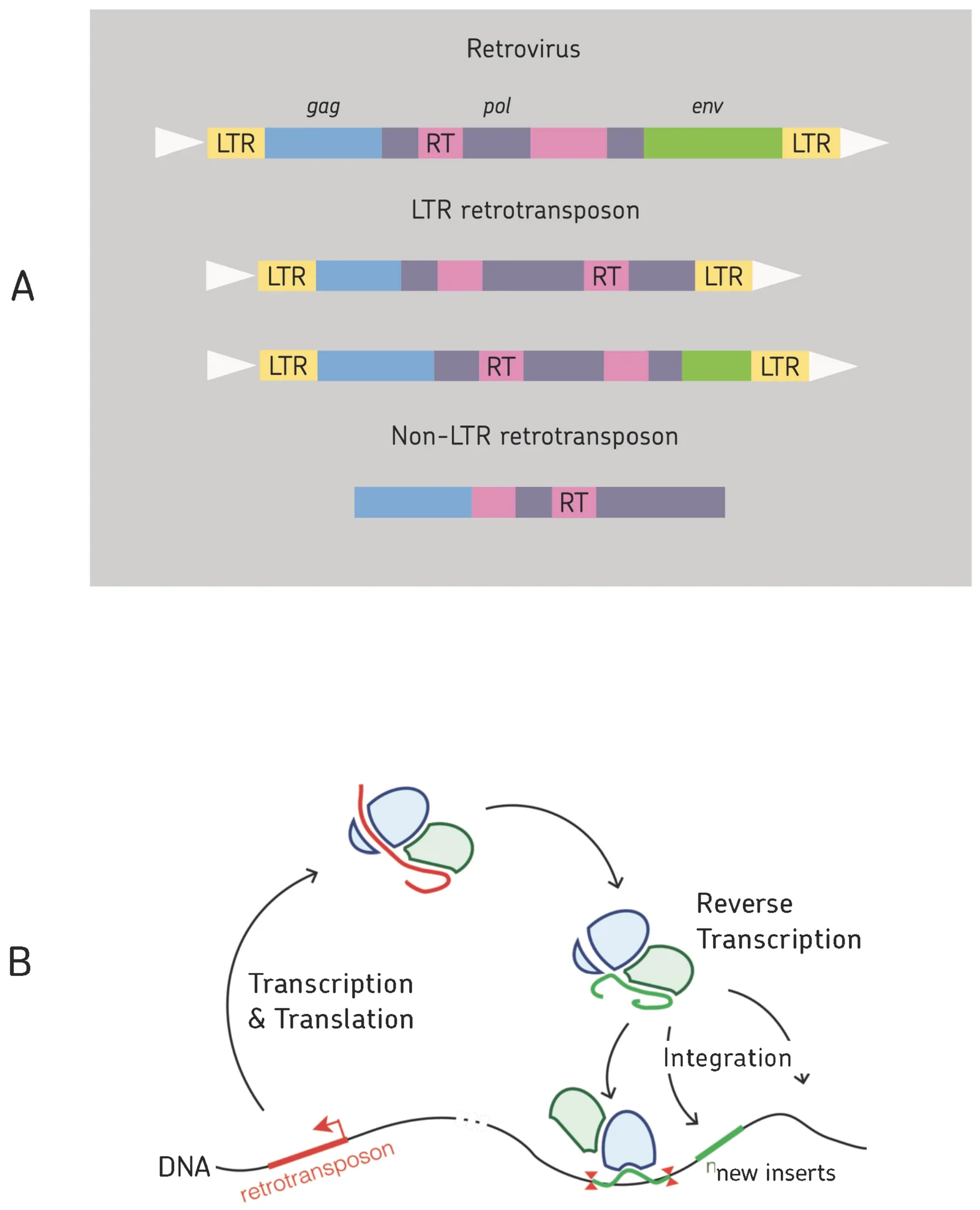
RNA retrotransposons. (**A**) Structures derived from retroviruses. Long-terminal repeats (LTRs) enclose the structural genes gag, pol, and env, as well as the genes from RNA retrotransposons. All RNA retrotransposons encode the gene for the enzyme reverse transcriptase (RT). (**B**) Life cycle of an RNA retrotransposon. Transcription and translation of the DNA-integrated retrotransposon lead to RNA (red) and RT protein production followed by transcription to DNA (green) and its integration into the host genome at a new insertion site. (**A**) is created by the author. (**B**) is reproduced from Wikimedia Commons under the respective Creative Commons or Public Domain licenses.

Interestingly, mimiviruses may be infected by virophages [30], and defend against them through the mimivirus virophage resistance element (MVRE) system [30]. Bacteria and bacteriophages present a similar “pas de deux” (dance for two).

In this section, only selected examples of RNA or DNA viruses are discussed. The great majority of viruses are not presented.

## 4. Bacteria

This section deals with the changes that occurred in the transition from an anaerobic to an aerobic world due to oxygenic photosynthesis. This section describes associated consequences such as changes in the oceans and atmosphere, as well as the creation of oxidative stress.

The earliest bacterium-like organisms proliferated in prehistoric times (about 4 to 2.5 billion years ago), giving rise to a great variety of forms. Some of these were capable of photosynthesis (about 2.7 billion years ago). C and S isotopic signatures of microbial metabolism about 3.5 billion years ago derived from microbial mats called thrombolites [1,2].

### 4.1. Photosynthesis with Production of Sugar and Oxygen

Photosynthesis consists of two phases, a light (photo energy-collecting) phase and a dark (biosynthetic) phase. Energy from the light phase is transferred via NADPH and ATP to fix CO_2_ from the atmosphere. In the dark phase, C from this CO_2_ is transformed into sugar (C_6_H_12_O_6_, glucose). Prokaryotes then use a process of anaerobic glycolysis to degrade glucose for metabolic purposes.

We do not know for certain when and how photosynthesis originated. There could have been processes of anoxygenic photosynthesis (e.g., green sulfur bacteria, purple bacteria) and oxygenic photosynthesis (e.g., cyanobacteria and their precursors). Energy for oxygenic photosynthesis is only taken from photons, while energy for anoxygenic photosynthesis also requires electrons. These electrons are generated by photosystem II (PS II) together with protons (H^+^) through the redox enzyme NAD glycohydrolase (NADase) and its reduction equivalent NADH dehydrogenase (NADHase). This reductive system helped (and still helps) to remove H from H_2_O molecules according to the formula H_2_O = O + 2 H^+^ + 2 e^−^ [1,2].

The sites of photosynthesis in cyanobacteria are thylakoids; in particular, grana thylakoids and stroma thylakoids are stacks of disks filled with chlorophyll. These thylakoids provide a huge surface area for photosynthesis [1]. The initial light reaction in thylakoids is exerted through an ETC involving, among other things, D1/D2 proteins of PS II and the peripheral protein PsbO, which stabilizes the O_2_-evolving complex. During ETC reactions, electrons move from H_2_O through PS II via lipid soluble plastoquinones to photosystem I (PS I) and finally to NADP^+^, which is reduced to NADPH. Protons are pumped from the stroma into the thylakoid lumen, creating an electrochemical proton gradient that drives ATP synthesis. The protons flow down this gradient through an ATP-synthase complex in the membrane and reenter the thylakoid stroma [1] (p. 108). NADPH enters the Calvin cycle in the stroma, which performs the dark reaction of photosynthesis.

Photosynthetic ETCs (PETCs) convert solar energy masterfully into the essential energy (ATP) and reducing power (NADPH) for all cellular activities [31]; this is true for both prokaryotes and eukaryotes. Algae perform photosynthesis in endosymbiogenesis-derived chloroplasts (see Section 6.1). A recent review focused on microalgal bioenergy production from the perspective of PETCs [25], and it has been concluded that PETCs drive two key bioenergetic vectors, as follows: direct hydrogen generation and indirect biomass synthesis [31].

Microbes have a fascinating repertoire of bioenergetic enzymes and a huge variety of ETCs to cope with very different environmental conditions, such as varying oxygen concentrations, electron acceptors, pH, and salinity [32]. Microbial ETCs use different redox reactions to induce redox potential differences (ΔE). While the standard membrane redox potential is 0 millivolts (mV) (25 °C, pH 7), bacterial redox enzyme-mediated reactions involving inorganic compounds can change redox potentials from −400 mV to + 800 mV [26]. Redox reactions performed by multienzyme complexes include reactions such as those involving 2H^+^/H_2_, SO_3_^2−^/H_2_S, ferrihydrite/Fe^2+^, NO_3_^−^/NH4^+^, Mn^4+^/Mn^2+^, and O_2_/H_2_O. Organic compounds also contribute to alterations in redox potential; for example, ferredoxin_(ox,red)_, NADP^+^/NADPH, NAD^+^/NADH, FAD^+^/FADH_2_, and Fe(III)citrate/Fe(II)citrate. Bacteria that use such redox reactions are obligate anaerobes (e.g., *Acetobacterium woodii*, *Geobacter sulfurreducens*), facultative anaerobes (e.g., *Escherichia coli*, *Shewanella oneidensis*), and aerobes (e.g., *Corynebacterium glutamicum*, *Pseudomonas aeroginosa*) [32].

### 4.2. Cyanobacteria

Bacteria and especially cyanobacteria ruled the Earth’s oceans unchallenged from 3 to about 1.5 billion years ago [1,2]. About 3.5 billion years ago, there existed non-photosynthetic lineages that are closely related to cyanobacteria; e.g., Margulisbacteria [33] and Melainabacteria [34]. The evolution of oxygenic photosynthesis was of great evolutionary significance [35], which starts with the oxidation of water to O_2_ through a light-driven reaction catalyzed by photosystem II (PS II) [35]. Apart from chlorophyll, cyanobacteria also contain accessory carotenoid pigments such as phycocyanines, phycoerythrines, and carotinoides. These pigments broaden the spectrum of light that can be absorbed, thus allowing photosynthesis to proceed under weak light conditions. Filamentous forms of cyanobacteria produced great floating mats on the ocean surfaces. While this long period of cyanobacterial dominance has been called the “age of blue-green algae,” the use of the term “algae” is somewhat misleading because it suggests a relationship with eukaryotic algae [2].

A redefinition of the cyanobacterial lineage has been proposed based on phylogenomic analysis of distantly related non-phototrophic lineages [36]. In particular, cyanobacteria have been defined as “Organisms in the domain bacteria able to carry out oxygenic photosynthesis with water as an electron donor and to reduce carbon dioxide as a source of carbon, or those secondarily evolved from such organisms” [36].

Cyanobacteria, like all prokaryotes, are capable of horizontal gene transfer (HGT) and recombination [37]. Their genomes and metagenomes exhibit high levels of genomic diversity, partly driven by gene flow within and across species. Processes such as recombination and horizontal transfer of novel genes are facilitated by the mobilome, which includes plasmids, TEs, and bacteriophages [37].

### 4.3. Oxidative Stress

Life had to adapt to an aerobic world, which resulted in oxidative stress. Oxidative and nitrogenic stress [38] result from the excessive intracellular accumulation of reactive free radicals, such as reactive oxygen species (ROS) and reactive nitrogen species (RNS). Such stress contributes to the formation of advanced glycation end products and, thereby, to deleterious structural modifications of proteins and nucleic acids. Cellular antioxidants (e.g., glutathione) and antioxidant enzymes (e.g., superoxide dismutase, catalase, and glutathione peroxidase) were developed to sequester ROS and RNS, thus maintaining an optimal balance of the cellular redox status [38].

### 4.4. Cyanophages

Cyanobacteria can be infected by nm-sized phages called bacteriophages. About 80% of bacteria from oceans are infected by such phages, which have coexisted with the bacteria for 3.5 billion years. On any given day, about 20% of the terrestrial biomass consists of bacteria lysed by bacteriophages. This material serves as food for phytoplankton such as algae, of which there are more than 5000 varieties in the sea. Thus, the bacteriophages—by lysing bacteria—facilitate evolution by accelerating the food chain [39].

The number of bacteriophages depends on the season. During autumn and winter, the phages in bacteria (cyanophages) change their mode of behavior. Instead of lysing their host, they become lysogenic; that is, they integrate into their DNA or remain as episomes (a special type of plasmid) in the cell and thereby persist. In spring and summer, when the cyanobacteria expand explosively to form large mats or biofilms (“blooms”), the phages become activated and turn into a lytic cycle [40]. Some blooms along coasts can be observed even from airplanes.

The first cyanophage (*LPP-1*, dsDNA virus) was isolated in the early 1960s and sequenced in 2023 [41]. Cyanophages are considered an important factor in the early evolution of oxygenic photosynthesis. Many of them carry psbA and psbD genes that encode D1 and D2 proteins, respectively, which are core proteins of the PS II system in oxyphototrophs that influence the proper functioning of oxygenic photosynthesis [42].

Interesting details have been reported regarding the symbiosis of a P2-family phage with the deep-sea bacterium *Shewanella putrefaciens* [43]. About one-third of shewanella isolates harbored a prophage at the 3′-end of the tmRNA (a hybrid between tRNA and mRNA). About 0.1% of host cells were lysed to release P2 constitutively during bacterial host growth. P2 phage production was induced by a prophage-encoded rep protein and its excision by the cox protein. P2 excision greatly hindered bacterial growth and biofilm formation [43].

As natural predators of cyanobacteria, cyanophages hold promise as eco-friendly agents against harmful cyanobacterial blooms. These blooms often produce cyanotoxins which pose risks to public health and ecosystems by contaminating surface waters and drinking water sources [44]. By engineering cyanophages, it may be possible to develop a highly specific control strategy [44]. A first step has recently been achieved; namely, CRISP/Cas12a-based genome editing of the cyanophage *Anabeana* sp. [45].

### 4.5. Deep-Sea Bacterial Communities

A metagenomic analysis of deep-sea bacterial communities has been performed [46]. The deep-sea environment is characterized by extreme conditions such as limited light, low oxygen levels, high pressure, and nutrient scarcity. Among the predominant phyla found at a depth of 1000 m, the top ten were Proteobacteria, Firmicutes, Bacteroidetes, Actinobacteria, Planctomycetes, Acidobacteria, Nitrospinae, verrucomicrobia, Candidatus Melainabacteria, and Cyanobacteria [46].

### 4.6. Mutualistic and Parasitic Interactions

Bacteria of the *Endozoicomonadaceae* family have been found in association with marine organisms across various ocean ecosystems [47]. Their interactions range from mutualistic to parasitic, depending on the host species and ecological context. Eubiotic relationships with corals and dysbiotic relationships (i.e., imbalance of microbial communities) with bivalves and fish have been reported [47].

### 4.7. Stromatolites and Thrombolites

Stromatolites and thrombolites represent accretionary structures formed in shallow water through the trapping, binding, and cementation of sedimentary grains by biofilms of microorganims, especially cyanobacteria [48]. Such structures also include viruses—in particular, phages—and represent one of the most abundant living entities in aquatic and terrestrial environments [48]. An analysis of phage communities of marine (Bahamas) and freshwater (Mexico) microbialites revealed biogeographical variability and suggested that they may have derived from an ancient community [48]. Stromatolites and thrombolites have existed on Earth from ancient times up to the present.

## 5. Archaea

Archaea are small cells without a nucleus which have been observed in bullet, rod, filament, or one of many other, sometimes bizarre shapes. In contrast to bacteria, archaea do not have a peptidoglycan-containing cell wall. They have a cell membrane without peptidoglycans and a cell wall, cytoplasm with ribosomes, circular plasmid DNA, and a chromosome in the nucleoid area (Figure 2).

### 5.1. Extremophiles

Of particular importance for archean life are extremophiles, which are able to adapt to extreme environments. Examples include thermophiles (heat-loving, 80 °C), cryophiles (cold-loving, −10 °C), halophiles (salt-loving), methanotrophs (which use methane for their metabolic needs, electrons, and carbon units), acidophiles (acid-loving, pH 3), and alkaliphiles (alkali-loving, pH 9) [1,2].

Halophilic archaea (*DPNAA*) of geothermally influenced salt lakes from Ethiopia have been shown to contain viruses and virus satellites with nested interactions [49]. These relationships involve three or more microbes, similar to a Russian doll arrangement. Head-tailed, tailless icosahedral, pleomorphic, and spindle-shaped viruses from these symbioses represented 16 different families, where these viruses exhibited convergent adaptation to hypersaline environments, encoded diverse auxiliary metabolic genes, and exchanged genes or mobile genetic elements (mobilomes) horizontally with each other [49]. The metagenome of a virus sample (metavirome) of a hypersaline environment in Spain was also highly diverse and differed from those of virus communities from marine and other aquatic environments. Putative hosts were the archaeon *Haloquadratum walsbyi* and the bacterium *Salinibacter ruber* [50]. Archaeal viruses show a stunning diversity of morphologies, with many resembling tailed bacterial phages.

### 5.2. Defense Mechanisms

An Asgard archaeal argonaute protein (PIWI HrAgo1) has been reported to play a crucial role in defense against viruses and transposons [51], which shares a common origin with eukaryotic PIWI proteins [51]. Argonaute proteins are the central effectors of RNA-guided RNA silencing pathways in eukaryotes [51]. Another recent study revealed that Asgard defense systems comprise 89 unique systems, including argonaute, NLR, Mokosh, viperin, Lassamu, and CBASS [52]. To counteract cell death induced by the bacterial cyclic oligonucleotide-based anti-phage signaling system (CBASS)—an innate immune system that induces cell suicide—phage genes encode immune evasion proteins [53].

Bacterial immune systems encode a large array of antiviral (antiphage) systems [52]. Recent investigations have identified unexpected similarities between the immune systems of bacteria and animals [54]. Proteins displaying sequence similarity with recently discovered antiphage systems have been found to be widespread in eukaryotes and maintain a role in human immunity [55]. The anti-transposon piRNA pathway is evolutionarily linked to the antiphage system Mokosh [55]. Human GTPases of immunity-associated proteins (GIMAPS) and two genes (FHAD1 and CTRC) are related to the Eleos and Lamassu prokaryotic systems, respectively, and exhibit antiviral activity [55].

CRISPR-Cas systems stand out as the only known RNA-programmed pathways for detecting and destroying bacteriophages and plasmids [56]. The binding, editing, and regulation of class 1 CRISPR-Cas effectors have been described [56].

Two vesicle secretion systems also concern defense and immunity. The bacterial type VI secretion system (T6SS) is widely distributed in Gram-negative bacteria and is similar to the puncturing device of bacteriophages [57]. A puncturing device is also employed by the complement system (CS) in animals [58], in which the multiple fragments of complement bind to specific receptors on innate and adaptive immune cells, the activation of which translates the initial humoral innate immune response into cellular innate and adaptive immunity [58]. Like other bacteria, alpha-proteobacteria generate their own extracellular vesicles (EVs)—a trait that was passed on to mitochondria, enabling them to generate mitochondria-derived vesicles (MDVs) [59]. MDVs are vesicles ranging from 30 to 200 nm in diameter which carry cargo for degradation by lysosomes and peroxisomes [59]; they are involved—among other things—in intercellular communication, induction of immune responses, and surface antigen presentation [59].

### 5.3. Asgard Archaeota

A complex type of archaea, designated as *Lokiarchaeota,* has been reported to encode eukaryotic signature genes (ESGs) and, thus, to bridge the gap between archaea and eukaryotes [60]. In particualr, these ESGs are suggestive of sophisticated membrane remodeling capabilities [60]. The structure and physiology of these so-called *Asgard* archeaota have been resolved by Japanese scientists via the culturing of cells from deep marine sediments together with two types of bacteria [61]. The cultured cell, *Prometheo-archaeum syntrophicum*, has octopus-like tentacles with which it makes contact with the co-cultured bacteria to enable the exchange of important molecules [61]. It is an anaerobic, extremely slow-growing, small coccus (around 550 nm in diameter) that degrades amino acids through syntrophy (cross-feeding) [61]. A model for eukaryogenesis, termed the entangle/engulf/endogenize model, was proposed [61].

Asgard archaeota shed light on the evolutionary origins of the eukaryotic ubiquitin/endosomal sorting complexes required for transport (ESCTR) machinery [60]. Many archaeal species possess homologues of ESCRT-III and Vps4—the components that execute the final membrane scission reaction [62]. Analyses of Asgard archaea have suggested a pre-eukaryotic origin for the ubiquitin-coupled ESCRT system and a likely path of ESCRT evolution via a series of gene duplication and diversification events [63].

The scientific discussion centers around the contributions of *Asgard* archaea, alpha-proteobacteria, and a possibly phagocytic last eukaryotic common ancestor (LECA) population. Extensive gene transfer from prokaryotes could have contributed to (i) the cytoskeleton system of micro- and intermediate filaments and microtubules, (ii) new endomembranes, (iii) oxidative phosphorylation (OXPHOS), (iv) microtubular flagella, (v) peroxisomes (detoxification), (vi) mitophagy (dismantling of old mitochondria for recycling), and (vii) mitotic and meiotic nuclear division.

### 5.4. Eukaryogenesis, FECA, and LECA

The process of eukaryogenesis evolved about 2.7 billion years ago. The first eukaryotic common ancestor (FECA) and the LECA lived in the Proterozoic, about 2.1 to 1.5 billion years ago. Radical steps have been proposed to have occurred along the way from the FECA to the LECA [64], involving a host related to present-day Asgard archaea and an endosymbiont related to present-day alphaproteobacteria. It was proposed that pre-symbiosis, efficient ATP generation, internal ROS formation, and enhanced retention of genes supplied by HGT facilitated this unique transformation [64]. The LECA already had the genetic machinery encoding the endomembrane apparatus, spliceosome, nuclear pore, and myosin and kinesin cytoskeletal motors [65]. A complex ubiquitin signaling system of archaeal origin enabled complex post-translational regulation [66].

### 5.5. Habitat Transition in the Evolution of Bacteria and Archaea

A study has reviewed the literature on habitat transitions among various bacterial and archaeal lineages [67], and it was reported that cells dependent on microbial hosts—particularly bacteria from the candidate phyla radiation—have undergone repeated habitat transitions from environmental sources into animal microbiomes. Comparisons were made between free-living cells, methanogenic archaea, cellular endosymbionts, and bacteriophages [67].

## 6. Eukaryotes

### 6.1. Endosymbiogenesis

The origin of eukaryotes occurred between 2.1 and 1.8 billion years ago. As a result of evolutionary forces, the organizational complexity of eukaryotes is much greater than that of prokaryotes. The American biologist Lynn Margulis was the first to suggest that eukaryotes did not arise from any single prokaryote, but were derived from a symbiosis event involving two or more types of bacteria [68]. Nuclei, chloroplasts, and mitochondria of eukaryotes each contain genes encoding rRNA [2]. Comparisons of the sequences of bases of these genes have shown that nuclear, chloroplast, and mitochondrial DNAs represent distinct evolutionary lineages. Chloroplasts are plastids coding for chlorophyll and are closest to cyanobacteria evolutionarily. In contrast, mitochondria are organelles which are closest to alpha-proteobacteria. While chloroplasts contain the enzymes required for photosynthesis, mitochondria contain enzymes required for oxidative metabolism (respiration). Therefore, a host cell that was able to accommodate such organelles would have had enormous evolutionary advantages.

Symbiogenesis played a major role in this important evolutionary period, creating so-called holobionts. If the two organisms (host and symbiont) involved in creating a holobiont are no longer capable of living independently from each other, it means that a new species has evolved. Unfortunately, these facts were not available to Darwin.

Early events in plastid evolution center around the question of how endosymbionts became organelles [69].

### 6.2. Chloroplasts of Algae and Photosynthesis

Only two eukaryotic membrane-enclosed endosymbionts are recognized as canonical organelles, as follows: the chloroplasts of algae and plants (biosynthetic factories) and mitochondria (bioenergetic and metabolic factories). Details about the mechanisms of photosynthesis involving the production of sugar and oxygen have been described for bacteria, particularly cyanobacteria. Chloroplasts of eukaryotes have been shown to utilize similar mechanisms. However, as a result of evolutionary forces, the organizational complexity and effectivity of photosynthesis in algae and plants are much higher than those in bacteria.

### 6.3. Mitochondria and Respiration

The first mitochondrial endosymbionts could have easily doubled the prokaryotic host’s available ATP pool, provided that (i) genes for growth on environmental amino acids were transferred from the mitochondrial symbiont to the archaeal host, and (ii) the host was an autotroph using the acetyl-CoA oxidation (Krebs cycle) pathway [70].

As key organelles involved in respiration for the production of energy and CO_2_, mitochondria are the same size as bacteria and are surrounded within the eukaryotic cell by a double membrane. The inner mitochondrial membrane (IMM) consists of lipids that are characteristic of bacteria (e.g., cardiolipin), while the outer mitochondrial membrane (OMM) consists of lipids and porin molecules characteristic of bacteria [71]. The IMM shows multiple infoldings. These so-called cristae are sites of ATP synthase activity. Like the thylakoids of chloroplasts, the cristae of mitochondria provide a huge surface area, thus optimizing energy production.

Between the IMM and the OMM, there is a space which is used to establish a proton gradient for the ATP synthase pump [71]. The IMM is equipped with multi-enzyme protein complexes of the ETC; this is the site where chemical bond energy from fuel substrates is trapped and transferred to ATP. The ETC of mitochondria consists of four protein complexes (I to IV) associated with the following redox enzymes: NADH ubiquinon oxidoreductase (I), succinate dehydrogenase (II), cytochrome c reductase (III), and cytochrome c oxidase (IV). Electrons from the coenzymes NADH and FAD_2_ are transported via these multienzyme complexes to O_2._ Electron energy is also used to push protons outward across the IMM; consequently, the proton gradient drives H^+^ inward through proton channels of the ATP synthase transmembrane protein. This important enzyme couples proton movement to ATP synthesis.

There is a kind of symbiotic relationship between two membrane transporters, the NADPH oxidase complex and voltage-gated proton channels (HV 1) [72]. In particular, NADPH oxidase moves electrons and HV 1 moves protons. Electron flux is electrogenic and rapidly depolarizes the membrane potential. Excessive depolarization can turn off electron transport via self-inhibition; however, this is prevented by proton flux that balances the electron flux [72].

Let us now examine some organic compounds of the ETC, in order to better understand their complexities. Plastiquinone (PQ) and ubiquinone (UQ) are two important prenylquinones functioning as electron transporters in the ETC involved in oxygenic photosynthesis and the aerobic respiratory chain [73]. PQ exists in plants, while UQ is widely present in plants, animals, and microbes. More than 35 enzymes are known to be involved in the biosynthesis of PQ and UQ [73]. Multi-heme cytochromes also play crucial role [74], as they are involved in electron transport and biological energy conversion. Microbes can acquire energy from the environment by extending their ETCs to external solid (e.g., metal) electron donors or acceptors. For example, organohalide-respiring bacteria use halogenated organic compounds as terminal electron acceptors [6]. Organohalide respiration is unique in that dehalogenase—a cobamine-containing iron/sulfur protein—is recruited as a terminal reductase in the ETC [6]. Another ion-motive electron transport complex in prokaryotes is the Rnf complex [75], which energetically couples the cellular ferredoxin to the pyridine nucleotide pool [73] and covers a redox range more negative than −320 mV [75].

An intermediate of mitochondrial aerobic metabolism is acetyl CoA. This two-carbon molecule is fed into the Krebs cycle with its OXPHOS capacity. CO_2_, a byproduct of the Krebs cycle, is released. Oxidation of acetyl-CoA in the Krebs cycle provides energized electrons to generate ATP; furthermore, acetyl-CoA is also a crucial intermediate in lipid metabolism [2]. O_2_ is involved at the very end of the ETC. Overall, the ETC produces 32 molecules of ATP and 6 molecules of H_2_O per glucose molecule. The mitochondrial proton turbines pump and produce 150 ATP reactions and molecules per second, comprising an energy-producing machine operating at astounding efficiency [71].

In the mitochondrion’s matrix, an oxidative system is used for respiration. With the help of cytochrome oxidase, it “burns” hydrogen into water, where electrons, protons, and oxygen are fused to produce H_2_O according to the formula 2 e^−^ + 2 H^+^ + O = H_2_O [2]. Interestingly, this formula is the reverse of that characterizing the activity of chloroplasts.

A master gene regulator arose, as follows: the transcription factor (TF) nuclear factor erythroid 2-related factor (Nrf2). This TF maintains cellular redox homeostasis and cytoprotective defenses [76]. Activation of Nrf2 is associated with scavenging of ROS, suppression of lipid peroxidation and ferroptosis (iron-dependent cell death), stabilization of mitochondrial function, and mitigation of apoptosis and endoplasmic reticulum (ER) stress [77].

### 6.4. Further Endosymbionts

Lynn Margulis has argued for further bacterial endosymbionts in addition to mitochondria and chloroplasts. The eukaryote flagella, cilia (locomotory structures), and even the spindle of mitosis have been suggested to be derived from certain kinds of bacteria, such as spirochaetes (Figure 2). Such merging of disparate organisms to produce evolutionary novel forms is called endosymbiogenesis.

Bioenergetic interactions between chloroplasts and mitochondria have been studied in the green alga *Chlamydomonas reinhardtii* [78], and inactivation by RNA interference of the ATP synthase subunit beta was reported to alter both mitochondrial and chloroplastic function and morphology [78].

Intracellular endosymbionts are widespread in unicellular and multicellular eukaryotes. Examples include (i) rhizobium (fungus) with bacteria, (ii) ciliate (protist) with *Chlorella* (green microalgae), (iii) amoeba *Paulinella* (protist) with cyanobacteria [79], (iv) *Trypanosomatid* (protist) with a bacterium [80], and (v) aphid (plant-sucking louse, heteroptera, multicellular) with *Buchnera* (gamma-proteobacterium).

### 6.5. Chromosomes, Nuclear and Cell Division

Eukaryoitic chromosomes are more complex than prokaryotic ones. The 3D structure of a double helix was discovered in 1953 by J. Watson and F. Crick in Cambridge (UK), based on an analysis of the X-ray structure from a DNA crystal produced by R. Franklin. This double helix explains how genetic information is stored, as well as how it is copied.

Microbes also evolved the cell cycle, which is associated with chromosome division, nuclear division, and cell division. Replication of DNA occurs during the interphase stage S (synthesis), while G_1_ and G_2_ refer to pre- and post-synthesis periods. After mitosis (nuclear division) and cytokinesis (cell division), the cell may enter an arrested quiescent stage known as G_0_ [2]. Using yeast (*Saccharomyces cerevisiae*) as a model, Sir Paul Nurse—the 2001 winner of the Nobel Prize in Physiology or Medicine—investigated the spatiotemporal orchestration of mitosis by the enzyme cyclin-dependent kinase (CDK) [81]. He discovered that the CDK control system operates within distinct regulatory domains in the nucleus and cytoplasm [81]. Cell cycle studies have also been performed in *Saccharolobus islandicus*—a model archaeon for which the cell cycle can be synchronized and controlled [82].

### 6.6. Feeding Methods

Protists use various types of phagocytosis as a feeding method. *Amoeba* surround their prey with pseudopodia. *Leidyopsis*, a flagellate living in the intestine of termites, forms pseudopodia and ingests wood chips. *Didinium*, a ciliate, feeds only on *Paramecium*, which it swallows through a temporary cytostome. *Codonosiga*, a sessile flagellate with a collar of microvilli, feeds on particles suspended in the water drawn through its collar by the beat of its flagellum [2] (pp. 678–682). Phagocytosis has also been observed in metazoa; for instance, in phagocytes of the reticuloendothelial system (RES).

### 6.7. Viral Impacts

The origin of the virome of the last eukaryotic common ancestor (LECA) could be traced back to a small set of bacterial—not archaeal—viruses [83], likely due to the bacterial origin of eukaryotic membranes. Eukaryogenesis most likely involved two endosymbiotic events in a syntrophic model; in particular, a bacterial host engulfed an Asgard archaean, thereby preventing archaeal viruses from entry owing to a lack of archaeal virus receptors on the external membranes [83].

Viral histone repeats branch phylogenetically between archaean and eukaryotic nucleosomes and display intermediate functions, as reported in studies on *Escherichia coli* bacteria [84]. Type II DNA topoisomersases of the family A (Topo IIAs) are present in bacteria and eukaryotes [85], but they have no orthologs in archaea. An in-depth phylogenetic analysis suggests that Topo IIA was probably acquired from an ancestral member of the *Nucleocytoviricota*, giant viruses, before the emergence of the LECA [85]. Interesting details have also been reported from mimiviruses [86]. A large fraction of mimivirus replication proteins, including polymerase B, clamp, and clamp loaders, have been found to be of eukaryotic origin [85]; as such, it is likely that eukaryotes and giant viruses coevolved. Jit et al. used the RNA polymerase subunit ß (RpoB) gene—a marker that is conserved across bacteria, archaea, eukarya, and giant viruses—to reconstruct early phylogenies [87]. Their results revealed that giant viruses clustered closely with eukaryotes [87].

### 6.8. Recapitulation of Early Events

The first eukaryotes were undoubtedly unicellular, and many were photosynthetic autotrophs using energy from sunlight.

Some eukaryotes evolved animal-like characteristics and gave rise to a variety of protist (protozoan) phyla with diverse phenotypes and characteristics [2]. The descendants of another line of eukaryotes—the green algae—eventually gave rise to multicellular plants [1]. Multicellular organisms (metazoa), however, arose only about 1 billion to 700 million years ago.

Eukaryotes have the capacity of intracellular transport via vesicles. Expression of ESGs enables the construction of dynamic intracellular cytoskeletons, which can function as “cables” for the transport of vesicles. The origin of ESGs goes back to Asgard archaea.

Potential steps in the evolution of eukaryotes over time are summarized in Figure 4.

## 7. Discussion

This review focuses on microbes which developed in prehistoric times (the archaeon eon) and their roles during the later evolution of protists (eukaryotes). The second part of this review deals with microbes as key players during the evolution of metazoa (multicellular organisms).

The time period of 4–1 billion years ago is difficult to imagine, as we are used to thinking in terms of shorter and more recent time episodes. The narrative of evolution presented here is therefore associated with uncertainties. We should be aware that a large majority of microorganisms have not yet been discovered. Therefore, the understanding we obtain from the organisms described must be considered incomplete.

Nevertheless, scientific research with new genetic tools has created a wealth of genomic data about ancient microbes, and geoarchaeology has provided evidence for the Earth’s history. In recent decades, both fields have contributed to a better understanding of key processes arising during the evolution of life on Earth.

There are good reasons why the author favors the primordial RNA world hypothesis. The facts presented are more stringent than the alternative hypotheses of minerals and proteins or of metabolic circles at the beginning of life. We should seek to prove or refute a Martian origin for life on Earth [4].

All viroids and animal viruses are obligate intracellular parasites, as they require a host cell to establish a productive infection. Thus, it could be considered that viroids and parasitic animal viruses might have emerged after eukaryotes. The author argues that this could be explained by habitat transition; some examples are presented in Section 5.5. Viroids or parasitic animal viruses might have existed in prehistoric times based on environmental sources, and changed later to become parasites of plants or animals.

The evolution of eukaryotes most likely occurred stepwise. In a prebiotic first period, versatile RNA molecules, proteins, DNA, viroids, and viruses developed, leading to the hypothetical last universal cellular ancestor (LUCA). This was followed by a second period, in which prokaryotes such as bacteria and archaea developed. Alpha-proteobacteria and Asgard archaea then paved the way for the formation of the hypothetical first eukaryotic common ancestor (FECA). A third period involving several possibly phagocytic events from prokaryotes could have contributed to the cytoskeleton system and to new endomembranes, among other things. Extensive gene transfers then led to the hypothetical last eukaryotic common ancestor (LECA). In the fourth period, eukaryotes finally developed.

Figure 4 shows a scheme depicting how microbes developed roughly 4–1 billion years ago. In this context, the following question can be asked: Did the RNA world, bacteria, and archaea coexist temporally? My answer is yes, as it is difficult to imagine strict separation lines. During transition periods (which were long), these forms of life likely coexisted; for instance, during the period from 4.2 to 3.9 billion years ago, the LUCA and LACA likely coexisted with previous forms of life while, in the period from 2.1 to 1.5 billion years ago, the FECA and LECA likely coexisted with previous forms of life.

Although the potential roles of RNA or DNA viruses in the evolutionary processes that led to the emergence of eukaryotes are likely, relevant evidence remains scarce. Phylogenetic and structural studies have revealed that *Nucleocytoplasmic* large DNA virus (NCLDV) RNA polymerase catalytic cores share deep evolutionary roots with eukaryotic counterparts, implicating prehistoric gene transfer that shaped the LECA [87]. The genomes of *Nucleo*cytoviruses frequently encode eukaryotic signature proteins (ESPs) such as RNA- and DNA-processing proteins, vesicular trafficking factors, cytoskeletal components, and proteins involved in ubiquitin signaling [88].

The VE hypothesis lends rather radical support for a decisive role of viruses in eukaryogenesis. It states that a virus initiated the eukaryogenesis process by colonizing an archaeal host to create a virocell, which reprogrammed its metabolism to support a viral factory. Subsequently, viral processes facilitated the entry of a bacterium into the archaeal cytoplasm which eventually became reprogrammed to support the viral factory. As the latter increased control of the consortium, the archaeal genome was lost, the bacterial genome was greatly reduced, and the viral factory eventually evolved into the nucleus [21]. The VE hypothesis proposes that eukaryotes originated as an emergent superorganism and did not evolve from the LUCA via descent with incremental modifications. Another study has revealed a role of Nucleocytoviricota viruses during eukaryogenesis [22]. My position is that viroids and viruses (including bacteriophages) played an important role in eukaryogenesis, including the LUCA.

Evidence demonstrating that bacteriophages played a role in eukaryogenesis is still scarce. However, as bacteriophages have existed for about 4 billion years, not only in prokaryotes but also in eukaryotes and metazoa up to humans (as covered in Part II of this review), my answer is that they very likely played a role in eukaryogenesis.

A prerequisite for evolution was the capacity of microbes to defend themselves against parasitic viruses. CRISPR/Cas is one of these systems which, in modern gene technology, is used for precise genome editing. As an example, this technique has been applied to target covalently closed circular DNA (cccDNA) and to achieve complete cure from chronic hepatitis B (CHB) [89]. This is a significant result as over 250 million people globally are living with CHB, which is responsible for 83% of deaths from viral hepatitis [89].

Other control systems evolved between bacteria and bacteriophages. The explosive growth of cyanobacteria is regulated via lysis by activated endogenous cyanophage prophages. Giant DNA mimiviruses have developed an MRSE system to resist virophages. Furthermore, the human gut contains a multitude of bacteria and bacteriophages; the latter, infecting only bacteria of the gut, likely represent a similar control system.

The driving forces of microbial development include nested interactions of three or more microbes, which facilitated convergent environmental adaptations by horizontally exchanging genes or through mobile genetic elements encoding auxiliary metabolic genes.

The most important driving forces behind the emergence of eukaryotic cells—around 1.5 billion years ago—were likely endosymbiogenesis events between two or more types of bacteria leading to new cellular organelles. This hypothesis, proposed by Lynn Margulis, was later supported by the results of base sequence comparisons of genes encoding rRNA; in particular, nuclear, chloroplast, and mitochondrial DNA were found to represent distinct evolutionary lineages.

The present view regarding the origin of eukaryotic cells includes at least three different cell types which contributed genes, plastids, or organelles; namely, (i) cyanobacteria; (ii) alpha-proteobacteria; and (iii) Asgard archaea. Notably, chloroplasts are plastids coding for chlorophyll, which are evolutionarily closest to cyanobacteria; mitochondria are organelles that are closest to alpha-proteobacteria; and the intracellular transport of vesicles required eukaryotic signature genes, which are evolutionarily closest to Asgard archaea.

The identification of these distinct, yet relevant cell types required sophisticated and long-term cell and molecular biological work, further driven by new molecular genetic technologies which provide a means to efficiently evaluate evolutionary relationships.

## 8. Future Directions

The roles of various microbes during evolution are a fascinating topic that has attracted increasing attention among scientists, with examples of relevant theories including the endosymbiogenesis hypothesis and the recent viral eukaryogenesis hypothesis. Whether there existed a last universal cellular ancestor (LUCA), and whether evolutionary steps were small and incremental or radical and large remain a matter of debate. As most ancient microbes have not yet been identified, a lot of future work will be required to fill this gap. Another future direction is research on the biodiversity crisis.

## Figures and Tables

**Figure 2:**
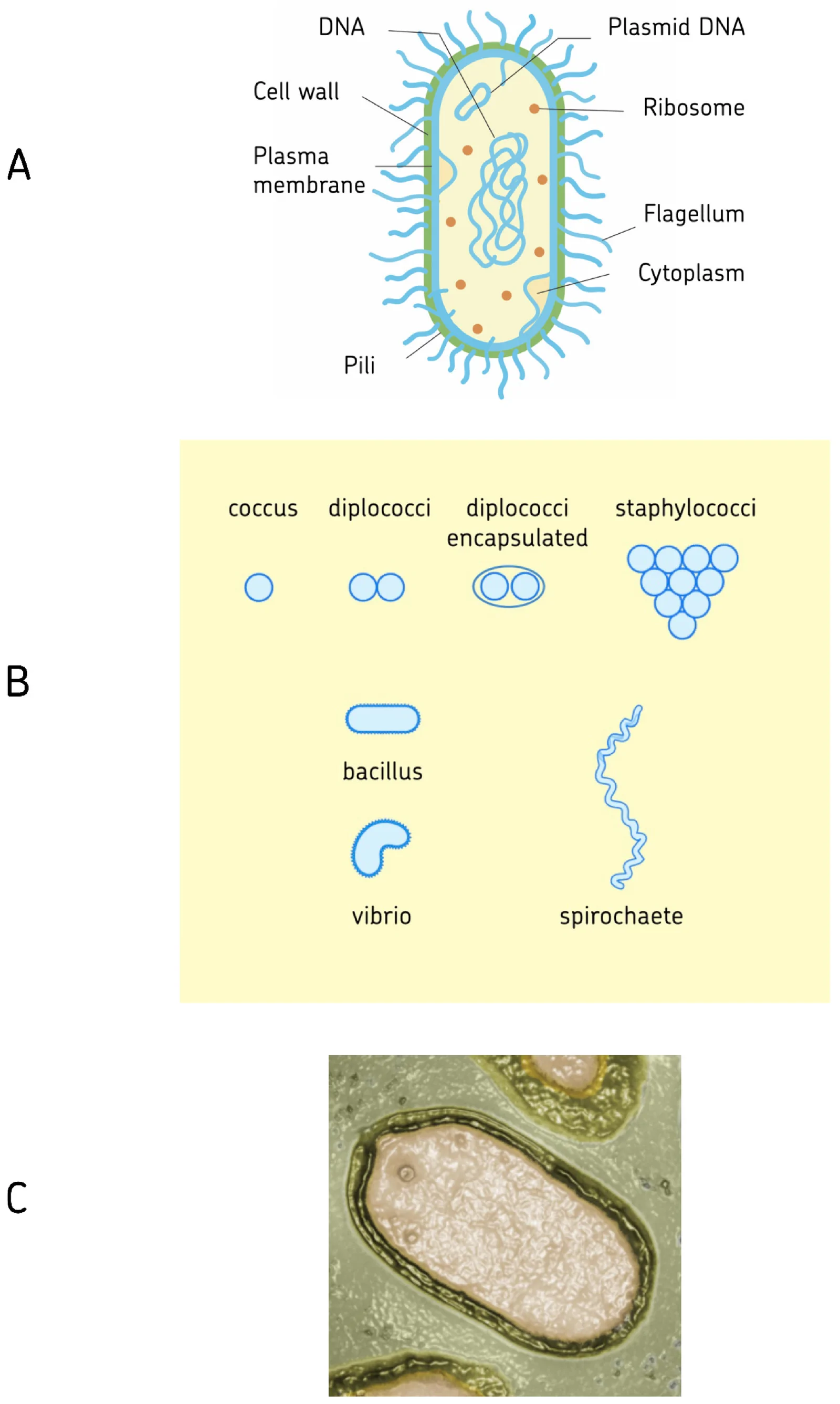
Biodiversity of microbes. (**A**) The structure of an archaeon, revealing two replicons: the central nucleoid with its chromosome, and plasmids. Further structures include ribosomes, the cytoplasm, a plasma membrane with flagella, and a cell wall with pili. (**B**) Morphological diversity of bacteria. (**C**) A colored electron microscopic picture of a giant virus (*Pandoravirus salinus*). (**A**) is based on content licensed from iStock and Shutterstock. (**B**,**C**) are reproduced from Wikimedia Commons under the respective Creative Commons or Public Domain licenses.

**Figure 4:**
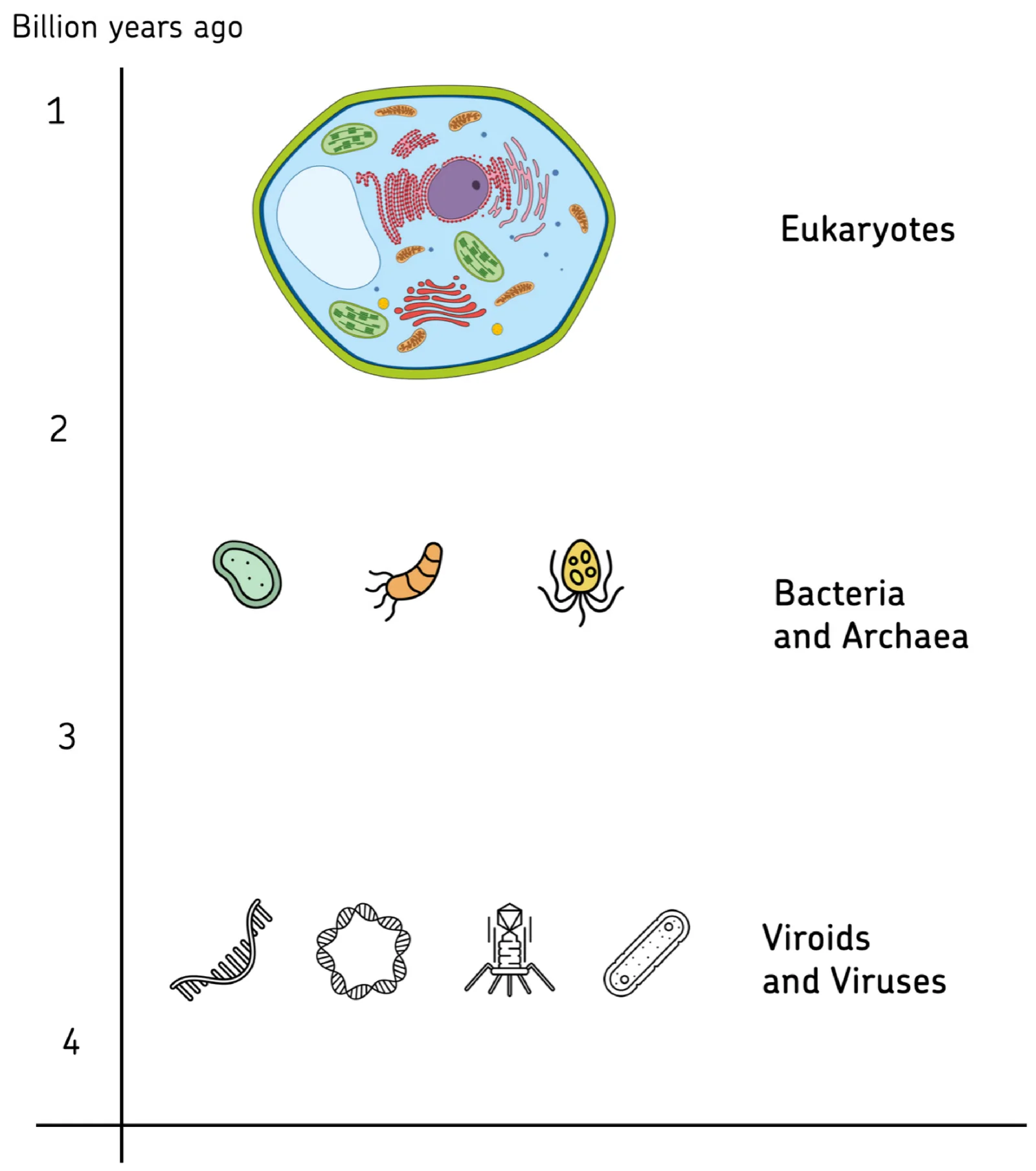
Potential steps in the evolution of eukaryotes. Bottom: viroids, plasmid DNA, phages, and viruses. Middle: bacteria and archaea. Three examples are shown: cyanobacteria (green), alpha-proteobacteria (orange), and Asgard archaea (yellow). Top: eukaryotes. This is an original infographic created by the author. It incorporates an adapted graphical element from licensed Shutterstock images and icons from The Noun Project.

## Data Availability

No new data were created or analyzed in this study. Data sharing is not applicable to this article.

## Abbreviations

A	adenine
AMP	adenosine monophosphate
ADP	adenosine diphosphate
ATP	adenosine triphosphate
ATPase	enzyme cleaving ATP
ATP synthase	ATP-producing enzyme
BIF	banded iron formation
bp	base pair
C	carbon
C	cytidine
CBAss	cyclic oligonucleotide-based anti-phage signaling system
cDNA	complementary DNA
cccDNA	covalently closed circular DNA
CDK	cyclin-dependent kinase
CHB	chronic hepatitis B
CH_4_	methane
Cas	CRISPR-associated genes
CO	carbon monoxide
CO_2_	carbon dioxide
CoA	coenzyme A
CRISPR	clustered regulatory interspaced short palindromic repeats
CS	complement system
CTP	cytosine triphosphate
DNA	deoxyribonucleic acid
dsDNA	double-stranded DNA
ER	endoplasmic reticulum
ESGs	eukaryotic signature genes
ETC	electron transport chain
EV	extracellular vesicle
FAD	flavin adenine dinucleotide
FECA	first eukaryotic common ancestor
GIMAP	GTPase of immunity-associated protein
G	guanine
GTP	guanine triphosphate
H	hydrogen
H_2_O	water
HGT	horizontal gene transfer
H_2_S	hydrogen sulfide
HV 1	voltage-gated proton channel
IMM	inner mitochondrial membrane
LACA	last archaeal common ancestor
LBCA	last bacteroid common ancestor
LECA	last eukaryotic common ancestor
LUCA	last universal cellular ancestor
MDV	mitochondrial-derived vesicles
Mn	manganese
mRNA	messenger RNA
MVRE	mimivirus virophage resistant element
mV	millivolt
N	nitrogen
NAD	nicotinamide adenine dinucleotide
NADase	NAD glycohydrolase
NADHase	NADH dihydrogenase
NADP	NAD phosphate
NADPH	reduced NADP
ncRNAs	non-coding RNAs
nm	nanometer
NH_3_	ammonia
NH_4_	ammonium ion
Nrf2	nuclear factor erythroid 2-related factor
O_2_	molecular oxygen
O_3_	ozone
OMM	outer mitochondrial membrane
OXPHOS	oxidative phosphorylation
P	phosphate
p	page
P_i_	inorganic phosphate
PQ	plastoquinone
PS	photosystem I or II of chloroplasts
PETCs	photosynthetic ETCs
RES	reticuloendothelial system
RNA	ribonucleic acid
RNAi	inhibitory RNA
rRNA	ribosomal RNA
RNS	reactive nitrogen species
ROS	reactive oxygen species
RT	reverse transcriptase
S	sulfur
SiO_2_	silica
ss	single-stranded
+ssRNA	positive single-stranded RNA
T	thymine
TE	transcriptional element
T6SS	type VI secretion system
TF	transcription factor
t-RNA	transfer RNA
tmRNA	hybrid between transfer and messenger RNA
U	uracil
UTP	uracil triphosphate
UQ	ubiquinone
